# Supplementary figures and images for: Molecular phylogeography reveals multiple Pleistocene divergence events in estuarine crabs from the tropical West Pacific
Source: PLoS One. 2022 Jan 13;17(1):e0262122. doi: 10.1371/journal.pone.0262122 (PMC8757990; doi:10.1371/journal.pone.0262122)

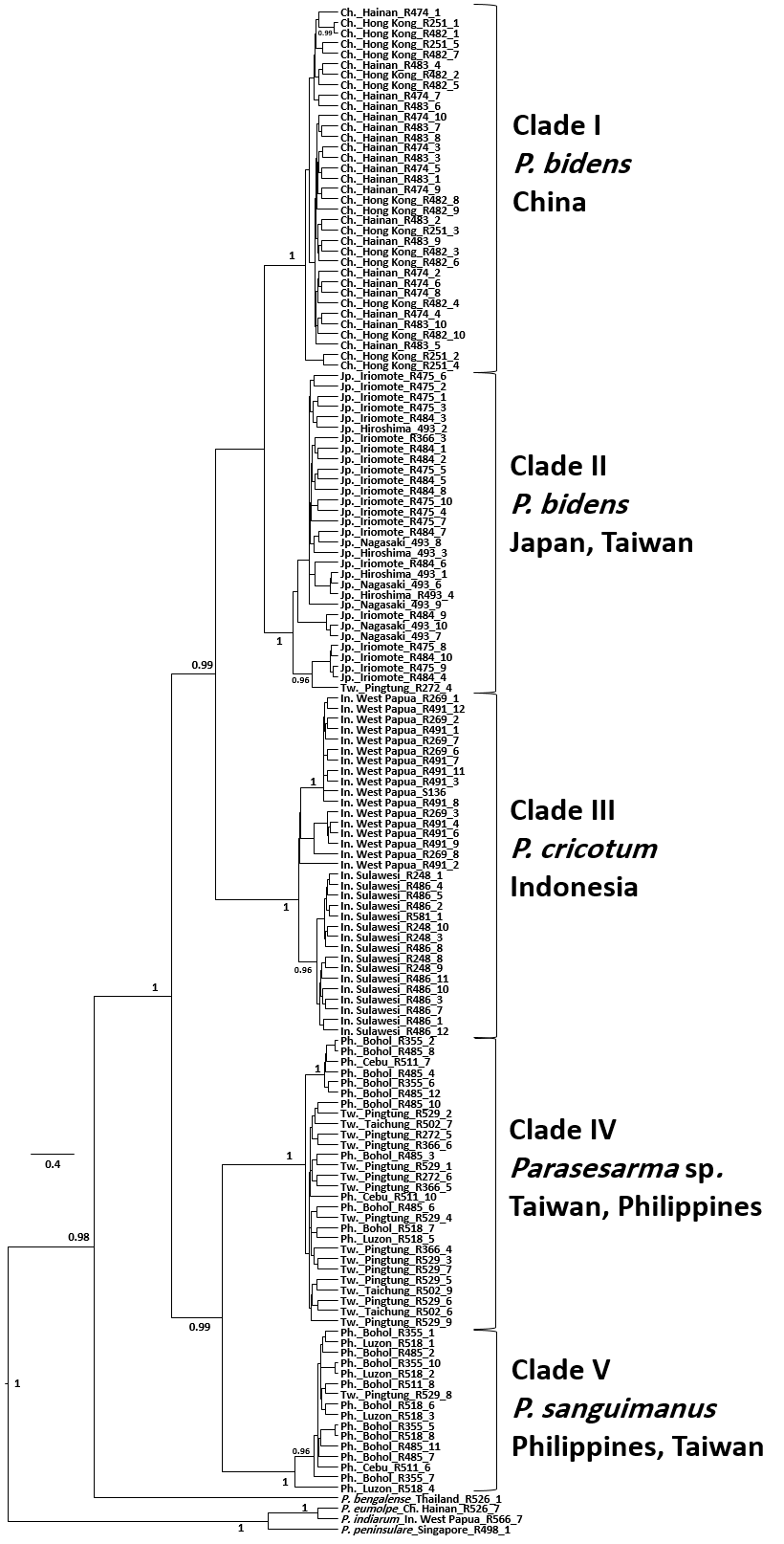

Supplement: S1 Fig — Values on tree branches refer to posterior probabilities in BI for each corresponding node. P. eumolpe, P. indiarum and P. peninsulare were selected as outgroups. Abbreviations: Ph, Philippines; Tw, Taiwan; Ch, China; Jp, Japan; In, Indonesia. (TIF) [file pone.0262122.s001.tif]

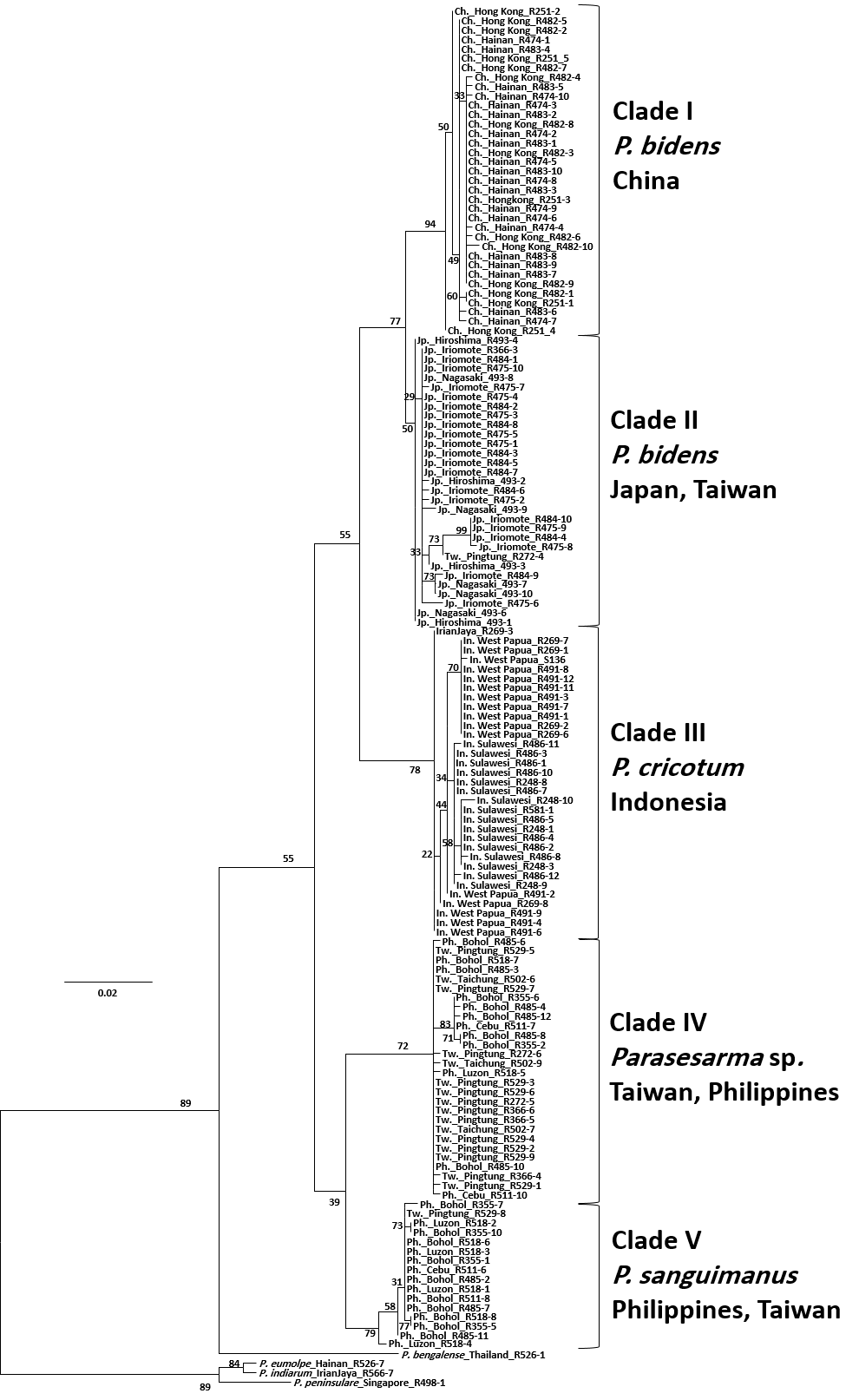

Supplement: S2 Fig — Numbers are bootstrap values after 1000 pseudoreplicates. P. eumolpe, P. indiarum and P. peninsulare were selected as outgroups. Abbreviations: Ph, Philippines; Tw, Taiwan; Ch, China; Jp, Japan; In, Indonesia. (TIF) [file pone.0262122.s002.tif]

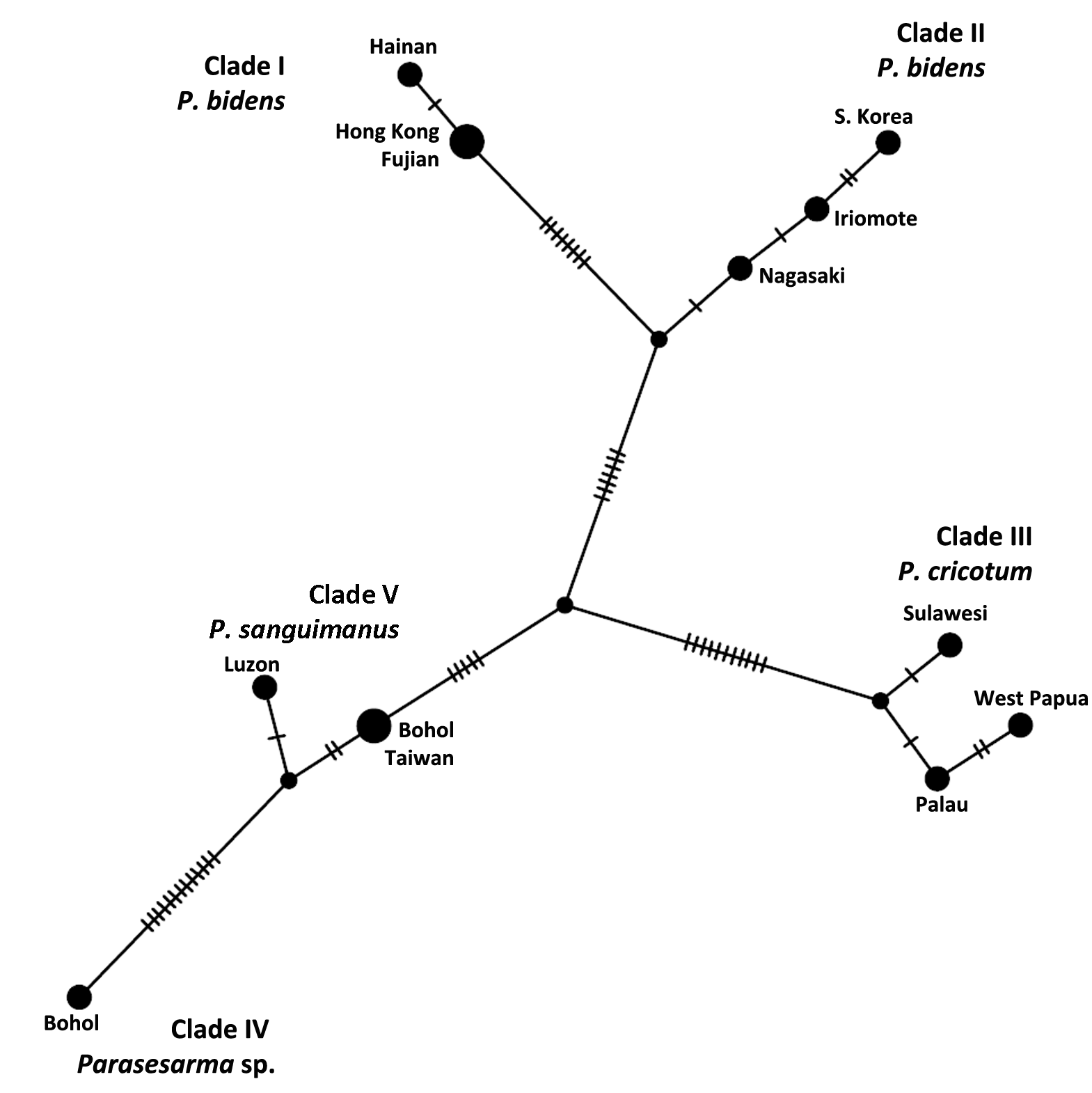

Supplement: S3 Fig — Hatch marks represent mutation steps. (TIF) [file pone.0262122.s003.tif]
