# Supplementary material for: Molecular phylogeography reveals multiple Pleistocene divergence events in estuarine crabs from the tropical West Pacific
Source: PLoS One. 2022 Jan 13;17(1):e0262122. doi: 10.1371/journal.pone.0262122 (PMC8757990; doi:10.1371/journal.pone.0262122)
Supplement: S1 Table — (DOCX) [file pone.0262122.s004.docx]

S1 Table. Material of *Parasesarma* examined for this study with locality, sex (M= male, F= female), size (maximum carapace width in millimeters), museum voucher number and year of collection, DNA extraction number and GenBank (NCBI) Accession number for COX1 (segments of the 5’end and the 3’ end, respectively) and 16S rRNA.

| Locality & coordinates | Species | Sex, size | Museum voucher, year of collection | DNA extraction numbers | Accession numbers | | |
| --- | --- | --- | --- | --- | --- | --- | --- |
|  |  |  |  |  | COX1 | | 16S |
|  |  |  |  |  | 5’end | 3’ end |  |
| Philippines, Bohol, Loboc River  9°35'56.8"N 124°0'35.5"E | *P. sanguimanus* | F, 16.0 | ZSM20210109, 2005 | R485-2 |  | OL619532 |  |
|  | *P. sanguimanus* | M, 24.3 | ZSM20210109, 2005 | R355-1 |  | OL619533 | OL677221 |
|  | *P. sanguimanus* | F, 18.3 | ZSM20210109, 2005 | R355-5 |  | OL619534 |  |
|  | *P. sanguimanus* | M, 17.8 | ZSM20210109, 2005 | R355-7 |  | OL619535 |  |
|  | *P. sanguimanus* | M, 16.0 | ZSM20210109, 2005 | R355-10 |  | OL619536 |  |
|  | *P. sanguimanus* | M, 14.9 | ZSM20210109, 2005 | R485-7 |  | OL619537 |  |
|  | *P. sanguimanus* | M, 14.5 | ZSM20210109, 2005 | R485-11 | KX400899 | OL619538 |  |
|  | *P. sanguimanus* | F, 14.4 | UF11433, 2005 | R511-8 |  | OL619539 | OL677221 |
|  | *P. sanguimanus* | F, 15.7 | UF11413, 2004 | R518-6 |  | OL619540 |  |
|  | *P. sanguimanus* | M, 12.0 | UF11415, 2006 | R518-8 |  | OL619541 |  |
|  | *Parasesarma* sp. | F, 24.1 | ZSM20210112, 2005 | R355-2 |  | OL619542 |  |
|  | *Parasesarma* sp. | M, 17.7 | ZSM20210112, 2005 | R355-6 |  | OL619543 |  |
|  | *Parasesarma* sp. | M, 16.6 | ZSM20210112, 2005 | R485-3 |  | OL619544 |  |
|  | *Parasesarma* sp. | F, 16.0 | ZSM20210112, 2005 | R485-4 | OL634849 | OL619545 | OL677223 |
|  | *Parasesarma* sp. | F, 15.6 | ZSM20210112, 2005 | R485-6 |  | OL619546 |  |
|  | *Parasesarma* sp. | F, 14.4 | ZSM20210112, 2005 | R485-8 |  | OL619547 |  |
|  | *Parasesarma* sp. | M, 14.5 | ZSM20210112, 2005 | R485-10 |  | OL619548 |  |
|  | *Parasesarma* sp. | M, 14.1 | ZSM20210112, 2005 | R485-12 |  | OL619549 |  |
|  | *Parasesarma* sp. | M, 12.9 | UF11414, 2005 | R518-7 |  | OL619550 |  |
| Philippines, Cebu, Matutinao River  9°48'36"N 123°22'04"E | *P. sanguimanus* | F, 16.8 | ZSM20210110, 2001 | R511-6 |  | OL619551 | OL677224 |
| Philippines, Cebu, Kawasan Falls  9°48'10"N 123°22'27"E | *Parasesarma* sp. | M, 20.2 | UF11356, 2005 | R511-7 |  | OL619552 | OL677225 |
|  | *Parasesarma* sp. | M, 20.6 | UF11311, 2005 | R511-10 |  | OL619553 |  |
| Philippines, Luzon, Subic Bay  14°46'51"N 120°16'51"E | *P. sanguimanus* | M, 16.3 | RUMF-ZC-7641, 2009 | R518-1 |  | OL619554 |  |
|  | *P. sanguimanus* | M, 15.3 | RUMF-ZC-7641, 2009 | R518-2 |  | OL619555 | OL677226 |
|  | *P. sanguimanus* | M, 12.1 | RUMF-ZC-7641, 2009 | R518-3 |  | OL619556 |  |
|  | *P. sanguimanus* | M, 12.1 | RUMF-ZC-7641, 2009 | R518-4 | KX400900 | OL619557 |  |
|  | *Parasesarma* sp. | F, 11.0 | RUMF-ZC-7642, 2009 | R518-5 |  | OL619558 |  |
| Taiwan, Pingtung, Hengchun | *P. sanguimanus* | NA | NCHUZOOL15525, NA | NA | LC510481* | NA |  |
| Taiwan, Pingtung, Paoli River  22°03'25"N 120°42'41"E | *P. bidens* | M, 17.4 | ZSM20210106, 1999 | R272-4 |  | OL619559 |  |
|  | *Parasesarma* sp. | F, 26.7 | ZSM20210113, 1999 | R272-5 |  | OL619560 |  |
|  | *Parasesarma* sp. | M, 23.2 | ZSM20210113, 1999 | R272-6 |  | OL619561 |  |
|  | *Parasesarma* sp. | M, 11.0 | ZSM20210113, 1999 | R366-4 |  | OL619562 |  |
|  | *Parasesarma* sp. | M, 13.8 | ZSM20210113, 1999 | R366-5 |  | OL619563 | OL677227 |
|  | *Parasesarma* sp. | F, 14.6 | ZSM20210113, 1999 | R366-6 |  | OL619564 |  |
|  | *Parasesarma* sp. | M, 20.3 | ZSM20210113, 1999 | R529-1 |  | OL619565 |  |
|  | *Parasesarma* sp. | M, 18.4 | ZSM20210113, 1999 | R529-2 |  | OL619566 |  |
|  | *Parasesarma* sp. | M, 17.8 | ZSM20210113, 1999 | R529-3 |  | OL619567 |  |
|  | *Parasesarma* sp. | M, 23.8 | ZSM20210113, 1999 | R529-4 |  | OL619568 |  |
|  | *Parasesarma* sp. | M, 19.6 | ZSM20210113, 1999 | R529-5 |  | OL619569 |  |
|  | *Parasesarma* sp. | M, 18.9 | ZSM20210113, 1999 | R529-6 |  | OL619570 |  |
|  | *Parasesarma* sp. | M, 18.8 | ZSM20210113, 1999 | R529-7 |  | OL619571 |  |
|  | *Parasesarma* sp. | M, 16.0 | ZSM20210113, 1999 | R529-9 |  | OL619572 |  |
|  | *P. sanguimanus* | M, 17.3 | ZSM20210111, 2011 | R529-8 |  | OL619573 |  |
| Taiwan, Taichung, Gaomei  No coordinates | *Parasesarma* sp. | M, 21.7 | NCHUZOOL 13393, 2006 | R502-6 |  | OL619574 |  |
|  | *Parasesarma* sp. | M, 20.3 | NCHUZOOL 13391, 1995 | R502-7 |  | OL619575 |  |
| Taiwan, Taichung, Wenliao  No coordinates | *Parasesarma* sp. | M, 19.9 | NCHUZOOL 13394, 2002 | R502-9 |  | OL619576 |  |
| China, Hainan, Wenchuang  19°33'21"N 110°49'25"E | *P. bidens* | M, 24.0 | RUMF-ZC-7643, 2010 | R483-1 |  | OL619577 |  |
|  | *P. bidens* | M, 15.5 | RUMF-ZC-7643, 2010 | R483-2 |  | OL619578 |  |
|  | *P. bidens* | M, 22.3 | RUMF-ZC-7643, 2010 | R483-3 |  | OL619579 |  |
|  | *P. bidens* | M, 14.2 | RUMF-ZC-7643, 2010 | R483-4 |  | OL619580 |  |
|  | *P. bidens* | M, 23.6 | RUMF-ZC-7643, 2010 | R483-5 |  | OL619581 |  |
|  | *P. bidens* | M, 22.2 | RUMF-ZC-7643, 2010 | R483-6 | OL634850 | OL619582 |  |
|  | *P. bidens* | M, 22.0 | RUMF-ZC-7643, 2010 | R483-7 |  | OL619583 |  |
|  | *P. bidens* | M, 19.6 | RUMF-ZC-7643, 2010 | R483-8 |  | OL619584 |  |
|  | *P. bidens* | M, 13.4 | RUMF-ZC-7643, 2010 | R483-9 |  | OL619585 |  |
|  | *P. bidens* | M, 21.0 | RUMF-ZC-7643, 2010 | R483-10 |  | OL619586 |  |
|  | *P. bidens* | M, 27.2 | ZSM20210105, 2010 | R474-1 |  | OL619587 | OL677228 |
|  | *P. bidens* | M, 25.6 | ZSM20210105, 2010 | R474-2 |  | OL619588 |  |
|  | *P. bidens* | M, 24.4 | ZSM20210105, 2010 | R474-3 |  | OL619589 |  |
|  | *P. bidens* | M, 22.7 | ZSM20210105, 2010 | R474-4 |  | OL619590 |  |
|  | *P. bidens* | M, 21.9 | ZSM20210105, 2010 | R474-5 |  | OL619591 |  |
|  | *P. bidens* | M, 18.9 | ZSM20210105, 2010 | R474-6 |  | OL619592 |  |
|  | *P. bidens* | M, 18.6 | ZSM20210105, 2010 | R474-7 |  | OL619593 |  |
|  | *P. bidens* | M, 17.3 | ZSM20210105, 2010 | R474-8 |  | OL619594 |  |
|  | *P. bidens* | M, 18.2 | ZSM20210105, 2010 | R474-9 |  | OL619595 |  |
|  | *P. bidens* | F, 17.1 | ZSM20210105, 2010 | R474-10 |  | OL619596 |  |
| China, Hong Kong, Mai Po  22°29'36.4"N 114° 2'2.4"E | *P. bidens* | M, 26.5 | ZSM20210104, 2009 | R482-1 | OL634851 | OL619597 | OL677229 |
|  | *P. bidens* | M, 24.4 | ZSM20210104, 2009 | R482-2 |  | OL619598 |  |
|  | *P. bidens* | M, 23.4 | ZSM20210104, 2009 | R482-3 |  | OL619599 |  |
|  | *P. bidens* | M, 21.9 | ZSM20210104, 2009 | R482-4 |  | OL619600 |  |
|  | *P. bidens* | M, 21.2 | ZSM20210104, 2009 | R482-5 |  | OL619601 |  |
|  | *P. bidens* | M, 21.7 | ZSM20210104, 2009 | R482-6 |  | OL619602 |  |
|  | *P. bidens* | M, 21.1 | ZSM20210104, 2009 | R482-7 |  | OL619603 |  |
|  | *P. bidens* | M, 20.4 | ZSM20210104, 2009 | R482-8 |  | OL619604 |  |
|  | *P. bidens* | M, 19.7 | ZSM20210104, 2009 | R482-9 |  | OL619605 |  |
|  | *P. bidens* | M, 16.9 | ZSM20210104, 2009 | R482-10 |  | OL619606 |  |
|  | *P. bidens* | M, 25.6 | ZSM20210104, 2009 | R251-2 |  | OL619607 |  |
|  | *P. bidens* | M, 25.3 | ZSM20210104, 2009 | R251-3 |  | OL619608 |  |
|  | *P. bidens* | M, 24.0 | ZSM20210104, 2009 | R251-4 |  | OL619609 |  |
|  | *P. bidens* | M, 24.1 | ZSM20210104, 2009 | R251-5 |  | OL619610 |  |
| China, Fujian | *P. bidens* | NA | NA | Hap2 | KM605220* | NA |  |
| Japan, Iriomote, Nadara River  24°23'52''N 123°49'54''E | *P. bidens* | M, 24.9 | ZSM20210101, 2010 | R484-1 |  | OL619611 |  |
|  | *P. bidens* | M, 25.2 | ZSM20210101, 2010 | R484-2 |  | OL619612 |  |
|  | *P. bidens* | M, 23.6 | ZSM20210101, 2010 | R484-3 |  | OL619613 |  |
|  | *P. bidens* | F, 22.9 | ZSM20210101, 2010 | R484-4 |  | OL619614 |  |
|  | *P. bidens* | M, 20.4 | ZSM20210101, 2010 | R484-5 |  | OL619615 |  |
|  | *P. bidens* | M, 21.9 | ZSM20210101, 2010 | R484-6 |  | OL619616 |  |
|  | *P. bidens* | M, 20.5 | ZSM20210101, 2010 | R484-7 |  | OL619617 |  |
|  | *P. bidens* | F, 18.6 | ZSM20210101, 2010 | R484-8 |  | OL619618 |  |
|  | *P. bidens* | F, 19.0 | ZSM20210101, 2010 | R484-9 |  | OL619619 |  |
|  | *P. bidens* | M, 18.5 | ZSM20210101, 2010 | R484-10 |  | OL619620 |  |
|  | *P. bidens* | M, 22.7 | RUMF-ZC-7644, 2010 | R475-1 |  | OL619621 | OL677230 |
|  | *P. bidens* | F, 18.8 | RUMF-ZC-7644, 2010 | R475-2 | KX761167* | OL619622 | KX761173 |
|  | *P. bidens* | M, 18.9 | RUMF-ZC-7644, 2010 | R475-3 |  | OL619623 |  |
|  | *P. bidens* | M, 19.7 | RUMF-ZC-7644, 2010 | R475-4 |  | OL619624 |  |
|  | *P. bidens* | F, 17.2 | RUMF-ZC-7644, 2010 | R475-5 |  | OL619625 |  |
|  | *P. bidens* | F, 16.0 | RUMF-ZC-7644, 2010 | R475-6 |  | OL619626 |  |
|  | *P. bidens* | F, 15.5 | RUMF-ZC-7644, 2010 | R475-7 |  | OL619627 |  |
|  | *P. bidens* | M, 15.6 | RUMF-ZC-7644, 2010 | R475-8 |  | OL619628 |  |
|  | *P. bidens* | leg | RUMF-ZC-7644, 2010 | R475-9 |  | OL619629 |  |
|  | *P. bidens* | F, 15.5 | RUMF-ZC-7644, 2010 | R475-10 |  | OL619630 |  |
| Japan, Ryukyu Islands, Iriomote  No coordinates | *P. bidens* | F, 16.8 | ZSM20210102, 2004 | R366-3 |  | OL619631 |  |
| Japan, Hiroshima, Kamo River, Takehara  34°19'39"N 132°53'51"E | *P. bidens* | M, 26.4 | ZSM20210103, 2010 | R493-1 |  | OL619632 |  |
|  | *P. bidens* | M, 23.7 | ZSM20210103, 2010 | R493-2 |  | OL619633 |  |
|  | *P. bidens* | F, 20.6 | ZSM20210103, 2010 | R493-3 |  | OL619634 |  |
|  | *P. bidens* | M, 19.9 | ZSM20210103, 2010 | R493-4 |  | OL619635 |  |
| Japan, Nagasaki, Taira River  32°49'32.2"N 129°46'59.3"E | *P. bidens* | F, 21.7 | RUMF-ZC-1337, 2010 | R493-6 |  | OL619636 |  |
|  | *P. bidens* | M, 20.6 | RUMF-ZC-1337, 2010 | R493-7 |  | OL619637 |  |
|  | *P. bidens* | M, 20.2 | RUMF-ZC-1337, 2010 | R493-8 | KX761166* | OL619638 |  |
|  | *P. bidens* | M, 19.2 | RUMF-ZC-1337, 2010 | R493-9 |  | OL619639 | OL677231 |
|  | *P. bidens* | M, 25.2 | RUMF-ZC-1337, 2010 | R493-10 |  | OL619640 |  |
| South Korea, Yellow Sea | *P. bidens* | NA | NA | NA | MT117066* | NA |  |
| Palau Island | *P. cricotum* | F, 13.1 | ZRC 2020.0263, 2019 | Si104B | MT502092* | NA |  |
| Indonesia, Sulawesi, Sg. Marisa  0°26'43"N 121°56'15"E | *P. cricotum* | M, 22.6 | ZRC 2000.1727, 2000 | R486-1 | MT502093* | OL619641 |  |
|  | *P. cricotum* | M, 21.4 | ZRC 2000.1727, 2000 | R486-2 |  | OL619642 |  |
|  | *P. cricotum* | M, 19.3 | ZRC 2000.1727, 2000 | R486-3 |  | OL619643 |  |
|  | *P. cricotum* | F, 18.4 | ZRC 2000.1727, 2000 | R486-4 |  | OL619644 | OL677232 |
|  | *P. cricotum* | F, 15.9 | ZSM20210108, 2000 | R486-5 |  | OL619645 |  |
|  | *P. cricotum* | M, 18.0 | ZSM20210108, 2000 | R486-7 |  | OL619646 |  |
|  | *P. cricotum* | F, 17.0 | ZSM20210108, 2000 | R486-8 |  | OL619647 |  |
|  | *P. cricotum* | F, 13.6 | ZSM20210108, 2000 | R486-10 |  | OL619648 |  |
|  | *P. cricotum* | F, 11.6 | ZSM20210108, 2000 | R486-11 |  | OL619649 |  |
|  | *P. cricotum* | F, 22.4 | ZSM20210108, 2000 | R486-12 |  | OL619650 |  |
|  | *P. cricotum* | F, 21.0 | RUMF-ZC-7645, 2000 | R248-1 |  | OL619651 |  |
|  | *P. cricotum* | F, 17.7 | RUMF-ZC-7645, 2000 | R248-3 |  | OL619652 |  |
|  | *P. cricotum* | M, 18.8 | RUMF-ZC-7645, 2000 | R248-8 |  | OL619653 |  |
|  | *P. cricotum* | M, 16.8 | RUMF-ZC-7645, 2000 | R248-9 |  | OL619654 |  |
|  | *P. cricotum* | M, 14.8 | RUMF-ZC-7645, 2000 | R248-10 |  | OL619655 |  |
| Indonesia, Sulawesi Tengah: Puna  1°25'1"S 120°40'56"E | *P. cricotum* | F, NA | ZRC2021.0883, 2000 | R581-1 |  | OL619656 |  |
| Indonesia, West Papua, Ajkwa  4°52'10"S 136°57'30"E | *P. cricotum* | M, 17.3 | ZRC2021.0884, 2000 | R269-1 |  | OL619657 |  |
|  | *P. cricotum* | M, 14.7 | ZRC2021.0885, 2000 | R269-2 |  | OL619658 |  |
|  | *P. cricotum* | F, 15.6 | ZRC2021.0886, 2000 | R269-3 |  | OL619659 |  |
|  | *P. cricotum* | M, 13.0 | ZRC2021.0887, 2000 | R269-6 |  | OL619660 |  |
|  | *P. cricotum* | M, 17.5 | ZRC2021.0888, 2000 | R269-7 |  | OL619661 |  |
|  | *P. cricotum* | M, 17.4 | ZRC2021.0889, 2000 | R269-8 |  | OL619662 |  |
| Indonesia, West Papua, Kamora  4°48'48"S 136°38'56"E | *P. cricotum* | M, 15.7 | ZSM20210107, NA | S136 |  | OL619663 | OL677233 |
|  | *P. cricotum* | M, 14.9 | ZRC 2021.0890, 2008 | R491-1 | KX400898* | OL619664 |  |
|  | *P. cricotum* | M, 15.8 | ZRC 2021.0891, 2008 | R491-2 |  | OL619665 |  |
|  | *P. cricotum* | M, 15.3 | ZRC 2021.0892, 2008 | R491-3 |  | OL619666 |  |
|  | *P. cricotum* | F, 15.7 | ZRC 2021.0893, 2008 | R491-4 |  | OL619667 |  |
|  | *P. cricotum* | F, 14.1 | ZRC 2021.0894, 2008 | R491-6 |  | OL619668 |  |
|  | *P. cricotum* | F, 13.8 | ZRC 2021.0895, 2008 | R491-7 |  | OL619669 |  |
|  | *P. cricotum* | F, 13.1 | ZRC 2021.0896, 2008 | R491-8 |  | OL619670 |  |
|  | *P. cricotum* | M, 12.5 | ZRC 2021.0897, 2008 | R491-9 |  | OL619671 | OL677234 |
|  | *P. cricotum* | F, 12.7 | ZRC 2021.0898, 2008 | R491-11 |  | OL619672 |  |
|  | *P. cricotum* | M, 11.8 | ZRC 2021.0899, 2008 | R491-12 |  | OL619673 |  |
| Thailand, Phuket | *P. bengalense* | M, 14.8 | SMF 49919, NA | R526-1 |  | OL619674 |  |
| Singapore, Mandai mangroves | *P. peninsulare* | M, 25.5 | ZRC 2017.1075, 2000 | R498-1 |  | OL619675 |  |
| China, Hainan | *P. eumolpe* | M, 23.5 | SMF 49922, 2010 | R526-7 |  | OL619676 |  |
| Indonesia, West Papua, Ajkwa | *P. indiarum* | M, 18.1 | ZRC 2000.1889, 2000 | R566-7 |  | OL619677 |  |

*Sequences recovered from GenBank (https://www.ncbi.nlm.nih.gov/).

NA: Not available for this study

UF: Florida Museum of Natural History, Florida, USA; NCHUZOOL: Zoological Collections of the Department of Life Science, National Chung Hsing University, Taichung, Taiwan; ZRC: Zoological Reference Collection of the Lee Kong Chian Natural History Museum, National University of Singapore; RUMF: Ryukyus University Museum, Fujukan, University of the Ryukyus, Okinawa, Japan; SMF: Forschungsinstitut und Museum Senckenberg, Frankfurt am Main, Germany; ZSM: Zoologische Staatssammlung, Munich, Germany.
