# Supplementary material for: Molecular phylogeography reveals multiple Pleistocene divergence events in estuarine crabs from the tropical West Pacific
Source: PLoS One. 2022 Jan 13;17(1):e0262122. doi: 10.1371/journal.pone.0262122 (PMC8757990; doi:10.1371/journal.pone.0262122)
Supplement: S2 Table — (DOCX) [file pone.0262122.s005.docx]

S2 Table. Primers used in the present study with corresponding DNA sequences (5´-3´) and references.

| Gene | Primer | Sequence | reference |
| --- | --- | --- | --- |
| COX1 | COL6 | TYTCHACAAAYCATAAAGAYATYGG | Schubart, 2009 |
|  | COL8 | GAYCAAATACCTTTATTTGT | Schubart, 2009 |
|  | COL1b | CCWGCTGGDGGWGGDGAYCC | Schubart, 2009 |
|  | COL11 | TCHGCWACHATAATTATTGC | Wyschetzki, 2012 |
|  | COH1b | TGTATARGCRTCTGGRTARTC | Schubart, 2009 |
|  | COH6 | TADACTTCDGGRTGDCCAAARAAYCA | Schubart & Huber, 2006 |
| 16S | 16L29 | YGCCTGTTTATCAAAAACAT | as “16L2” in Schubart et al., 2001 |
|  | 16H10 | AATCCTTTCGTACTAAA | Schubart, 2009 |

**References**

Schubart CD, Cuesta JA, Rodríguez A. (2001) Molecular phylogeny of the crab genus *Brachynotus* (Brachyura: Varunidae) based on the 16S rRNA gene. Hydrobiologia. 2001;449:41–46.

Schubart, C. D., & Huber, M. G. J. (2006). Genetic comparison of German populations of the stone crayfish, *Austropotamobius torrentium* (Crustacea: Astacidae). *Bulletin Français de la pêche et de la Pisciculture*, 380–381:1019–1028.

Schubart, C. D. (2009). Mitochondrial DNA and decapod phylogenies: the importance of pseudogenes and primer optimization. In: Martin, J. W., Crandall, K. A., & Felder, D. L., eds. Decapod Crustacean Phylogenetics. *Crustacean Issues*. CRC Press, Boca Raton, Florida, USA, 18, 47–65.

Wyschetzki, K. von. (2012). Revision of the *Perisesarma bidens* species complex (Decapoda: Brachyura: Sesarmidae) based on molecular and morphological data. *Unpublished Diplom thesis*, University of Regensburg, 1–120.
