# Supplementary material for: Molecular phylogeography reveals multiple Pleistocene divergence events in estuarine crabs from the tropical West Pacific
Source: PLoS One. 2022 Jan 13;17(1):e0262122. doi: 10.1371/journal.pone.0262122 (PMC8757990; doi:10.1371/journal.pone.0262122)
Supplement: S3 Table — (DOCX) [file pone.0262122.s006.docx]

S3 Table. Mean K2P distances (expressed in %) based on 618 bp of the 3’ end of COX1 gene between five phylogenetic clades recovered in the present study and calculated with MEGA version X.

|  | Clade I  *P. bidens* China | Clade II  *P. bidens*  Japan | Clade III  *P. cricotum*  Indonesia | Clade IV  *Parasesarma* sp.  Philippines, Taiwan | Clade V  *P. sanguimanus*  Taiwan, Philippines |
| --- | --- | --- | --- | --- | --- |
| Clade I |  | **1.4** | **2.7** | **3.8** | **3.4** |
| Clade II |  |  | **2.4** | **3.5** | **2.6** |
| Clade III |  |  |  | **4.0** | **3.9** |
| Clade IV |  |  |  |  | **2.7** |
