# Supplementary material for: Molecular phylogeography reveals multiple Pleistocene divergence events in estuarine crabs from the tropical West Pacific
Source: PLoS One. 2022 Jan 13;17(1):e0262122. doi: 10.1371/journal.pone.0262122 (PMC8757990; doi:10.1371/journal.pone.0262122)
Supplement: Attachment — Submitted filename: Comments from reviewers and answers.docx [file pone.0262122.s007.docx]

Journal requirements:

2. In your Methods section, please provide additional location information, including geographic coordinates for the data set if available.

**Answer.** For those localities that the data were available, the geographic coordinates are added in the S1 Table in the revised version.

3. In your Methods section, please provide additional information regarding the permits you obtained for the work. Please ensure you have included the full name of the authority that approved the field site access and, if no permits were required, a brief statement explaining why.

If no permits were required, please include the following statement:

'No permits were required for the described study, which complied with all relevant regulations.'

**Answer.** We are working on this issues and will be addressed upon acceptance.

4. In your manuscript, please provide additional information regarding the specimens used in your study. Ensure that you have reported specimen numbers and complete repository information, including museum name and geographic location.

**Answer.** We are working on this issues and will be addressed upon acceptance.

6. We note that Figure 1 in your submission contain map images which may be copyrighted. All PLOS content is published under the Creative Commons Attribution License (CC BY 4.0), which means that the manuscript, images, and Supporting Information files will be freely available online, and any third party is permitted to access, download, copy, distribute, and use these materials in any way, even commercially, with proper attribution. For these reasons, we cannot publish previously copyrighted maps or satellite images created using proprietary data, such as Google software (Google Maps, Street View, and Earth). For more information, see our copyright guidelines: <http://journals.plos.org/plosone/s/licenses-and-copyright>.

In the figure caption of the copyrighted figure, please include the following text: “Reprinted from [ref] under a CC BY license, with permission from [name of publisher], original copyright [original copyright year].”

 b. If you are unable to obtain permission from the original copyright holder to publish these figures under the CC BY 4.0 license or if the copyright holder’s requirements are incompatible with the CC BY 4.0 license, please either i) remove the figure or ii) supply a replacement figure that complies with the CC BY 4.0 license. Please check copyright information on all replacement figures and update the figure caption with source information. If applicable, please specify in the figure caption text when a figure is similar but not identical to the original image and is therefore for illustrative purposes only.

The following resources for replacing copyrighted map figures may be helpful:

Natural Earth (public domain): <http://www.naturalearthdata.com/>

**Answer:** As suggested here, we have replaced the map in the Fig. 1 with a new map from Natural Earth (public domain): <http://www.naturalearthdata.com/>, which complies with the CC BY 4.0 license. Accordingly, the caption for the Fig. 1 also is corrected now in the revise version (Lines 120-123).

**Fig 1. Sampling localities.** Localities for materials of the present study (solid black squares) and distribution map of the five clades (from Fig 2B) comprised within the *P. bidens* complex, with percentages of each clade in each locality of the study. The map reprinted from Natural Earth (public domain) [http://www.naturalearthdata.com/] under a CC BY license.

7. Please review your reference list to ensure that it is complete and correct. If you have cited papers that have been retracted, please include the rationale for doing so in the manuscript text, or remove these references and replace them with relevant current references. Any changes to the reference list should be mentioned in the rebuttal letter that accompanies your revised manuscript. If you need to cite a retracted article, indicate the article’s retracted status in the References list and also include a citation and full reference for the retraction notice.

**Answer**. The reference of Klaus et al. (2010) (reference number in previous format = 51) was identified irrelevant and deleted from the manuscript as commented by Reviewer #2.

Based on the comments from the reviewers we have added some more references to our manuscript. The reference numbers in the new version are: 12, 15, 43, 57, 60, 65, 72, 77, 80, 81, 84, 88, 89, 90, 95, 96, 97, 98, 99, and 100.


Reviewers' comments:

Reviewer #1: This manuscript examines the phylogeography of the mangrove crabs Perisesarma in the West Pacific. The result is clear and straightforward and should be published after some minor revision.

The MS is well written and the only aspects for improvement is it lacks references that are concerning on the phylogeography of mangrove organisms in the West Pacific for comparative purposes in the discussion. The authors consider phylogeography of intertidal species as a whole example. In fact, mangroves and rocky shores species may have different phylogeography patterns, because the local environment and currents in mangroves are much slower that rocky shore species. There are several major examples of phylogeography pattern of mangrove organisms which should be take into account for comparisons in the discussion. This includes:

1)Introduction: Lines 48, authors stated to state some factors to affect phylogeography of intertidal species. The formation of Isthmus of Panama have affect the genetic differentiation of Pacific and Caribbean crab populations (land mass mechanisms). The authors should cite some references concerning this mechanisms on mangrove crabs, including fiddler crabs:
Thurman CL, Alber RE, Hopkins MJ, Shih HT. 2021. Morphological and genetic variation among populations of the fiddler crab Minuca burgersi (Holthuis, 1967) (Crustacea: Brachyura: Ocypodidae) from shores of the Caribbean Basin and western South Atlantic Ocean. Zool Stud 60:19. doi:10.6620/ZS.2021.60-19.

**Answer**. We thank you for introducing this relevant study. In the new version, we cited this study through the manuscript (Introduction, lines 48, 50, Discussion line 446).

2)In addition to land mass mechanisms, some species can perform trans-Pacific dispersal mechanisms and resulted in some degree of genetic differentiation. This should also be stated in the introduction.
Trans-Pacific mechanisms
Hongjamrassilp W, Murase A, Miki R, Hastings PA. 2020. Journey to the west: trans-Pacific historical biogeography of fringehead blennies in the genus Neoclinus (Teleostei: Blenniiformes). Zool Stud 59:9. doi:10.6620/ZS.2020.59-09

**Answer**. The point is added now to the introduction (lines 50-52) and the study is cited (reference number 15).

3)Below are some phylogeography of mangrove organisms in the West Pacific and I would suggest the authors to cite and use their results to compare with the pattern in Perisesarma, as these organisms almost share similar habitats.
Mangrove mudskippers:
Chen, W., Hong, W. S., Chen, S. X., Wang, Q., & Zhang, Q. Y. (2015).
Population genetic structure and demographic history of the mudskipper
Boleophthalmus pectinirostris on the northwestern pacific coast.
Environmental Biology of Fishes, 98(3), 845–856.
Mangrove snails:
Kojima, S., Hayashi, I., Kim, D., Iijima, A., & Furota, T. (2004). Phylogeography
of an intertidal direct-developing gastropod Batillaria cumingi around
the Japanese Islands. Marine Ecology Progress Series, 276, 161–172.
Kojima, S., Kamimura, S., Iijima, A., Kimura, T., Mori, K., Hayashi, I., & Furota,
T. (2005). Phylogeography of the endangered tideland snail Batillaria
zonalis in the Japanese and Ryukyu Islands. Ecological Research, 20(6),
686–694.
Kojima, S., Kamimura, S., Kimura, T., Hayashi, I., Iijima, A., & Furota, T. (2003).
Phylogenetic relationships between the tideland snails Batillaria flectosiphonata
in the Ryukyu Islands and B. multiformis in the Japanese
Islands. Zoological Science, 20(11), 1423–1433.
Mangrove barnacles:
Chang, Y. W., J. S. M. Chan, R. Hayashi, T. Shuto, L. M. Tsang, K. H. Chu and B. K. K. Chan* (2017). Genetic differentiation of the soft shore barnacle Fistulobalanus albicostatus (Cirripedia: Thoracica: Balanomorpha) in the West Pacific. Marine Ecology-An Evolutionary Perspective, 38(2): e12422.
Mangrove crabs:
Wong, K. J. H., Chan, B. K. K., & Shih, H. T. (2010). Taxonomy of the sand bubbler crabs Scopimera globosa De Haan, 1835, and S. tuberculate Stimpson, 1858 (Crustacea: Decapoda: Dotillidae) in East Asia, with description of a new species from the Ryukyus, Japan. Zootaxa, 2345, 43–59.

**Answer**. We thank for introducing these relevant studies. They were indeed helpful and we have used them to add some more points and examples in our discussion (lines 335, 440,441). We have also cited them in our introduction (line 87). Reference numbers: 43,80,89, 90.

4)In the discussion, lines 438, the authors consider Philippines may be the origin of the clades that disperse northward in the West Pacific (sharing of genotype between Taiwan and the Philippines). Similar observation and conclusion was also addressed in the intertidal barnacle Hexechamaesipho and, Bresidium and Sesarmop crabs.
Please cite and refer to this example:
Tsang, L. M., B. K. K. Chan, G. A Williams and K. H. Chu (2013). Who is moving where? Molecular evidence reveals patterns of range shift in the acorn barnacle Hexechamaesipho pilsbryi in Asia. Marine Ecology Progress Series, 488: 187-200.
Li JJ, Shih HT, Ng PKL. 2020. The Taiwanese and Philippine species of the terrestrial crabs Bresedium Serène and Soh, 1970 and Sesarmops Serène and Soh, 1970 (Crustacea: Decapoda: Brachyura), with descriptions of two new species. Zool Stud 59:16. doi:10.6620/
ZS.2020.59-16.
Ng PKL, Li JJ, Shih HT. 2020. What is Sesarmops impressus (H. Milne Edwards, 1837) (Crustacea: Brachyura: Sesarmidae)? Zool Stud 59:27. doi:10.6620/ZS.2020.59-27.
**Answer**. We thank you for introducing the interesting works. In the new version we have used and cited (ref no. 84) the study done by Tsang et al. (2013) in the discussion (lines 408, 429, 469). The other two study are valuable taxonomic works, but they did not discuss phylogeography and population genetic.

5)Part of the above reference already show some of the glacial refugia. For more details on the glacial refugia in the West Pacific, the authors should refer:
Wu, T. H., Tsang, L. M., Chan, B. K. K., & Chu, K. H. (2015). Cryptic diversity
and phylogeography of the island-associated barnacle Chthamalus
moro in Asia. Marine Ecology, 36(3), 368–378.
Tsang, L. M., Chan, B. K. K., Wu, T. H., Ng, W. C., Chatterjee, T., Williams,
G. A., & Chu, K. H. (2008). Population differentiation in the barnacle
Chthamalus malayensis: Postglacial colonization and recent connectivity
across the Pacific and Indian Oceans. Marine Ecology Progress Series,
364, 107–118.
Chan, B. K. K., Tsang, L. M., & Chu, K. H. (2007). Morphological and genetic
differentiation of the acorn barnacle Tetraclita squamosa (Crustacea, Cirripedia) in East Asia and description of a new species of Tetraclita. Zoologica Scripta, 36(1), 79–91.

**Answer**. These studied are cited in the new version of the manuscript. The two studies, Wu et al. (2015) (ref no. 77) and Tsang et al. (2008) (ref no. 72), are cited for the glacial refugia (Discussion lines 321,330, 450). The study done by Tsang et al. (2008) and the work of Chan et al. (2007) (ref no. 95) are cited for another discussion, the separation between Taiwan and China mainland (Discussion lines 330, 450).

Reviewer #2: This is a study focusing on the phylogeography of the Parasesarma bidens species complex from West Pacific. While the methods of analyses were adequate, some biogeographical explanations based on the limited sample size should be more conservative.
Major comments:
* The included clades of this complex might be problematic. According to the trees in Shahdadi & Schubart (2017), Li et al. (2019), and Shahdadi et al. (2020), 3 spp. (bengalense, capensis, guttatum) in the Indian Ocean probably belong to the major clade of the bidens complex and at least closely related with the complex. The authors should include sequences of the above 3 spp. and might need to rewrite the part of "Phylogeny, phylogeography and evolutionary history" in Discussion.

**Answer**. We thank you for the nice comment. In the revised version we have added a sequence of *P. bengalense* as representative of the western clade (*P. bengalense*, *P. capensis* and *P. guttatum*), and reanalyzed the data. We made then a slight change in the method section (Materials & Methods, lines 157-160). We had slight changes in the results (new trees and Results lines 218, 229), and we made minor changes in the discussion according to the new results (Discussion lines 341-351). According to the trees in the abovementioned studies (Shahdadi & Schubart, 2017, Li et al., 2019 and Shahdadi et al., 2020) as well as in Shahdadi et al., 2021 (<https://doi.org/10.1071/IS20046>), this species group consists of two separate clades, the western clades (within the Indian Ocean) and the eastern clade (West Pacific). So the western species (*P. bengalense*, *P. capensis* and *P. gutttum*) form a monophyletic group, separated from the eastern species (*P. bidens*, *P. cricotum* and *P. sanguimanus*). During the time we were revising our manuscript, it was not possible for us to get material of all those western species in order to get the corresponding sequences, but we were able to add sequences of *P. bengalense* to our analyses. According to their monophyly, we believe that *P. bengalense* can represent the western clade and show the phylogenetic relationship between these two groups (eastern and western) (briefly discussed in the new version, Discussion, lines 341-351). As the new results (new trees) show, all five eastern clades stay under a bigger clade as a monophyletic group, separated from *P. bengalense*. After adding *P. bengalense* to our analyses as representative of the western clade, we are now able to calculate the divergence time (an estimate) between western and eastern clades. We therefore added a few sentences in this regards to our new version as addressed above.

we hope this change cover the issue, and the referee accept the manuscript in the present form, otherwise we need more time to ask for the material of *P. guttatum* and *P. capensis* and try to get new genetic data.

* L391, L399, L438-440: As the authors also mentioned that large sample size from Taiwan and Philippines are needed (L440-441), it is apparently too early to say one area could be considered as the origin. Otherwise, it will be more convincing by citing related references that Philippines is the origin of some marine species.

**Answer**. In the new version of our manuscript we have tried to soften this conclusion and we have given a relevant reference with the same conclusion (ref no. 84). At the end, however, we have admitted that larger sample size could give stronger support for the idea (see Discussion lines 469,470).

* L428-430: There might be barriers between Taiwan and China for some crab species, however populations in both areas of some species might show identical. For example, Austruca lactea (Shih, H.-T., Kamrani, E., Davie, P. J. F. & Liu, M.-Y., 2009. Genetic evidence for the recognition of two fiddler crabs, Uca iranica and U. albimana (Crustacea: Brachyura: Ocypodidae), from the northwestern Indian Ocean, with notes on the U. lactea species-complex. Hydrobiologia 635: 373-382) and some Metaplax spp. (Shih, H.-T., Hsu, J.-W., Wong, K. J. H. & Ng, N. K. 2019. Review of the mudflat varunid crab genus Metaplax (Crustacea, Brachyura, Varunidae) from East Asia and northern Vietnam. ZooKeys 877: 1-29). It might be necessary to discuss the larval behavior/ecology of related organisms and the ocean currents in this region (e.g. Taiwan Strait).

**Answer**. We thank you for this comment and in the new version we mentioned this diversity in phylogeographic structure and the possible reasons using these and others references (Discussion lines 446-456) (ref numbers 42, 44, 72, 95,96,97,98, 100).

Other comments:
* L30, L103: COX1 and 16S ?

**Answer**. Corrected now (line 30, 105).

* L95: "The latter species": It's clear to use the species name here.

**Answer**. Corrected now (line 95).

* L149: [51] is a study of freshwater crabs with only 16S and H3. I will suggest the authors check all the references and see if they are really relevant.

**Answer**. The reference 51 is deleted now from the manuscript. We have made other changes in the references in order to keep the relevancy and use the latest relevant studies.

* L15-156: It is better to use species from the sister clades as outgroup. See "major comments #1".

**Answer.** In the revised version of the manuscript, as suggested in the major comment 1, we have added a representative of the sister clade (*P. bengalense*) as one of the ingroups, in order to 1. Check their phylogenetic relationship, 2. To show monophyly of the eastern clade (Western Pacific clade) and 3. To calculate divergence time between eastern and western (within the Indian Ocean) clades (addressed in response to the major comments #1.

* L160: Will suggest to use the newest version of MEGA.

**Answer**. In the revised version we have used MEGA X to calculate the K2P distances (line 166) and the reference is also updated (ref no. 60). The slight changes in the values are applied in the table S5 for the new version of the manuscript.

* L173-174: Try to use formal citation and reference for "Microsoft Excel (2013)".

**Answer**. Corrected now (ref no. 65).

* L175: ARLEQUIN => ARLEQUIN 3.5; and delete "3.5" in L182.

**Answer**. Corrected now.

* L184 and others: Japan is composed by several large and small islands, but could not be considered as "mainland"! Will suggest to use "main islands of Japan" instead.

**Answer**. Corrected now.

* L194-196: It is confused that ".... (COX1) 16 sequences (including those from GenBank) with a length of 610 basepairs.....". Just say how many sequences you have from this study and how many sequences from GenBank. Rewrite this sentence.

**Answer.** To clarify and avoid confusion in the new version we have slightly changed the sentence (lines 200-203).

* L229: It should be confused whether it is necessary to provide 2 haplotype networks of COX1? One single tree of COX1 should be enough to provide strong evidence of the 5 clades and their relationship.

**Answer**. It is true that the haplotype networks confirm presence of five clades as the trees do. We, however, present the main haplotype network (for the 3’ end) as extra evidence for the clades showing the number of mutation steps between clades. It also gives visual evidence for haplotype relationship within each clade, which cannot be extracted from the trees. The haplotype network for the barcode region (for the 5’ end) is presented as a supplementary material in order to show the positions of the material from areas that we were not able to cover, but the data are available in GenBank. In order to avoid confusion we have tried to be clear in the Method section, as well as clarify the reason and our aim for presenting each illustration. So for this stage we prefer to keep both networks and, hoping that you accept our MS in this format.

* L325: If the authors did not run the test of rate constancy or similar tests, it is not adequate to say that the substitute rate is "a relatively precise time estimate", compared with the relaxed method (fossil data and other geological evidences" (L322-323).

**Answer**. It is true, and we have slightly change our state on this matter (Lines 324-325)

* L346: "major East Asian island" => "major islands in eastern Asia", because the Philippines is not a part of "East Asia".

**Answer**. Corrected now.

* L440: samples size => sample size

**Answer**. Corrected now.

* L393: Provide citation for "melting pot".

**Answer**. We see it as a fact and common term, so we see no need to add refrence.

* L425-428: For freshwater discharge in eastern Americas, I might suggest cite two relevant papers: (1) Thurman, C. L. , Hopkins, M. J., Brase, A. L. & Shih, H.-T. 2018. The unusual case of the widely distributed fiddler crab Minuca rapax (Smith, 1870) from the western Atlantic: an exemplary polytypic species. Invertebrate Systematics 32(6): 1465-1490. (2) Thurman, C. L, Alber, R. E., Hopkins, M. J. & Shih, H.-T. 2021. Morphological and genetic variation among populations of the fiddler crab Minuca burgersi (Holthuis, 1967) (Crustacea: Brachyura: Ocypodidae) from shores of the Caribbean Basin and western South Atlantic Ocean. Zoological Studies 60: 19.

**Answer**. We thank you for introducing the studies. In the new version, we have included more discussion and references (including Thurman et al., 2021) for this part (Discussion line 439-446) (reference numbers 12, 80, 89, 90, 91, 94).

* Fig. 1: It will be clear for readers to mark the names for the major localities on the map.

**Answer**. In the revised version names for the major localities are added in this figure.

* Fig. 2: A separated BI tree with all the haplotype names is suggested to be included in supporting information (similar to S3 Figure for ML tree). "P. Sanguimanus" => "P. sanguimanus".

**Answer**. In the new version we have added the BI tree as a supplementary figure (S3Fig). *P. sanguimanus* is also corrected now.

* S1 Table: Full names of museums for the abbreviation should be provided. In "size", all values in CW should be round to one decimal place. "14,47"=>"14.5". "Honlong" => "Houlong". BTW I found several haplotypes in S1 Table were not appeared in S3 figure! Need to check them carefully.

**Answer**. In the new version: Full names of museums for the abbreviation are added at the bottom of the S1 Table; values for the sizes are rounded; the spellings are checked and corrected now; the haplotypes in S1 Table that were not appeared in S3 figure are deleted now.
