# Supplementary material for: Molecular phylogeography reveals multiple Pleistocene divergence events in estuarine crabs from the tropical West Pacific
Source: PLoS One. 2022 Jan 13;17(1):e0262122. doi: 10.1371/journal.pone.0262122 (PMC8757990; doi:10.1371/journal.pone.0262122)
Supplement: Attachment — Submitted filename: Referee comments and answers.docx [file pone.0262122.s008.docx]

Referee comments and answers.

Dear Dr. Shahdadi,

Thank you for submitting your manuscript to PLOS ONE. After careful consideration, we feel that it has merit but does not fully meet PLOS ONE’s publication criteria as it currently stands. Therefore, we invite you to submit a revised version of the manuscript that addresses the points raised during the review process.

Dear authors,

Most of the comments were addressed. But I noticed that in the references list of your MS, most of the author name Chan, BKK was mis-spelled as Chan BK. Please correct all the Chan BK into Chan BKK in the reference list.

Answer.

We thank you for your kindness and the comment. We have corrected the mis-spelled names (Chan BK) in our new version (Chan BKK).

With best regards,

Adnan Shahdadi
